# Supplementary figures and images for: Epidemiological trends in alcoholic cardiomyopathy burden: A 32-year global and Chinese analysis (1990–2021) with projections to 2036
Source: PLoS One. 2025 Nov 18;20(11):e0336033. doi: 10.1371/journal.pone.0336033 (PMC12626323; doi:10.1371/journal.pone.0336033)

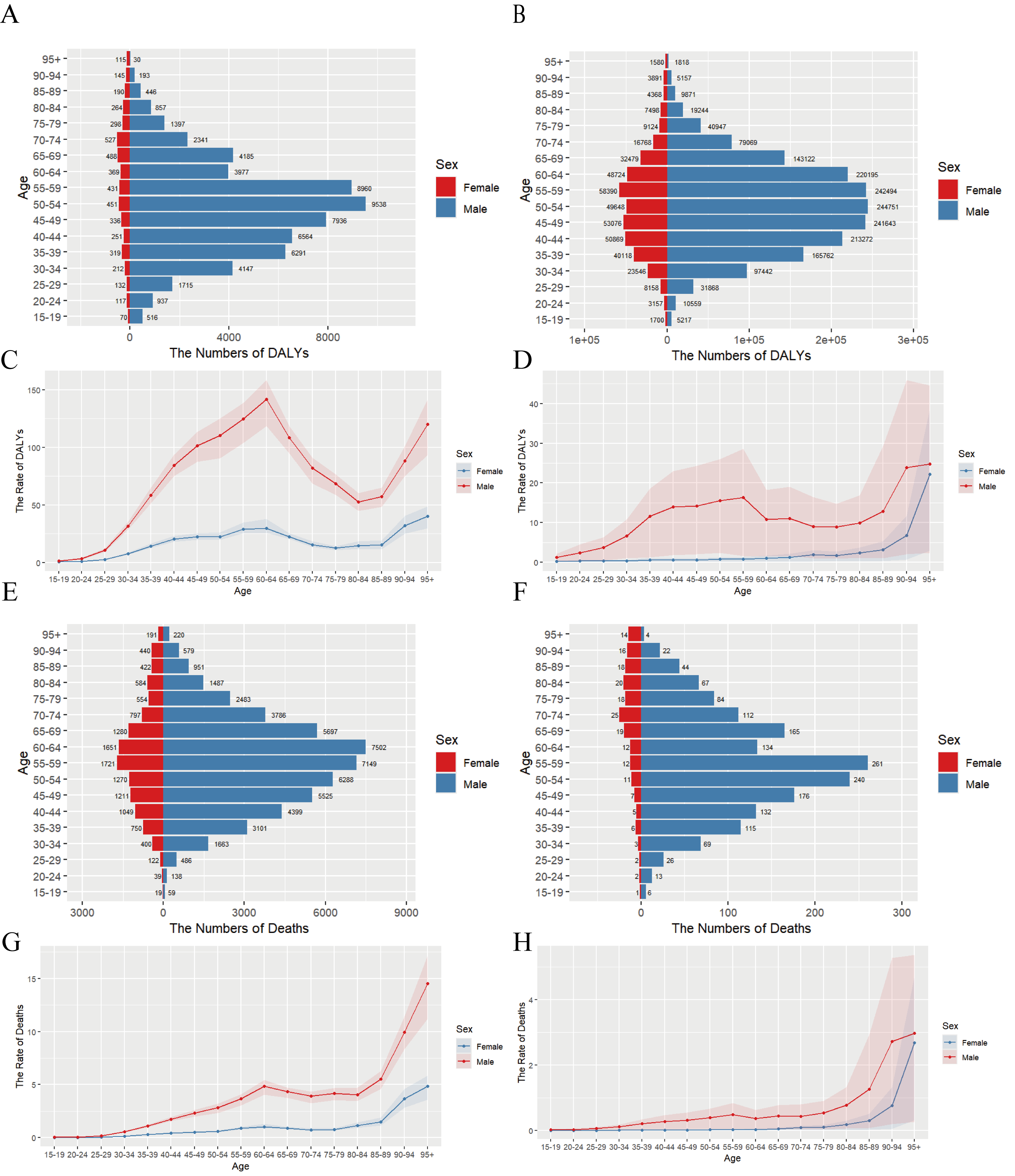

Supplement: S1 Fig — A Global age-specific morality numbers. B China age-specific deaths numbers C Global crude morality rates. D China crude morality rates; Global and China age-specific numbers and crude DALY rates of ACM in 2021. E Global age-specific DALYs numbers. F China age-specific DALYs numbers . G Global crude DALY rates. H China crude DALY rates. (PNG) [file pone.0336033.s001.png]

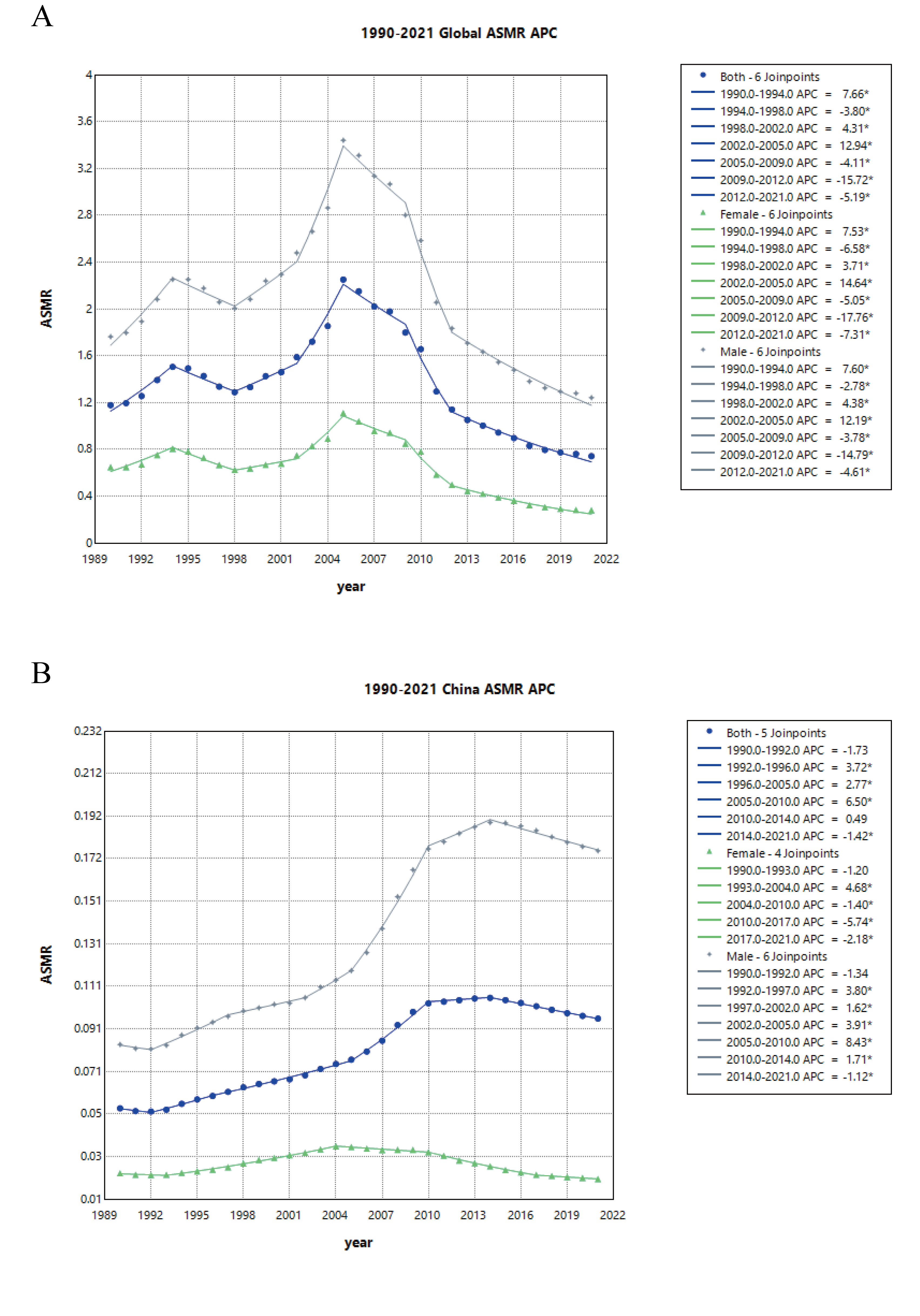

Supplement: S2 Fig — Global analysis; B China’s analysis. (PNG) [file pone.0336033.s002.png]
