# Supplementary material for: Epidemiological trends in alcoholic cardiomyopathy burden: A 32-year global and Chinese analysis (1990–2021) with projections to 2036
Source: PLoS One. 2025 Nov 18;20(11):e0336033. doi: 10.1371/journal.pone.0336033 (PMC12626323; doi:10.1371/journal.pone.0336033)
Supplement: Attachment — Submitted filename: response.docx [file pone.0336033.s010.docx]

Dear Editor:

Thank you for giving us the opportunity to revise and improve our manuscript entitled “***Epidemiological trends in alcoholic cardiomyopathy burden: A 32-year global and Chinese analysis (1990–2021) with projections to 2036***” (Manuscript Number: [PONE-D-25-11641R1]). We sincerely appreciate the time and effort that the reviewers and editorial team have dedicated to evaluating our work. Their constructive comments have been invaluable in enhancing the quality and clarity of our manuscript. We have carefully addressed all the concerns raised by **Reviewers**, and the revised manuscript now incorporates the following key modifications:

**Reviewers#1**

1. **Abstract**

**1.1 The background lacks a strong rationale for why ACM is a critical issue, beyond general disease burden. Clarify why ACM is a growing concern.**

- Added critical epidemiological paradox: “Despite global cardiovascular mortality declines, ACM burden continues rising paradoxically in middle-high income countries like China (200.4% case increase 1990-2021), revealing gaps in current prevention strategies”.
- Emphasized clinical significance: “Characterized by delayed clinical onset, ACM typically manifests as irreversible heart failure in middle age (45-69 years), creating missed opportunities for early intervention”.
- Highlighted public health urgency: “ACM remains preventable yet clinically overlooked, disproportionately affecting chronic alcohol users through alcohol-induced myocardial damage.”

**1.2 The results section could be more concise—it currently repeats statistics instead of synthesizing the main findings. Summarise key findings concisely.**

- Consolidated global trends: “Globally, ACM burden declined significantly (22.5-37.1% decrease in age-standardized rates), while China experienced a 200.4% case increase with rising mortality/disability rates.”
- Streamlined demographic analysis: “Three key disparities persist: (1) Male predominance (male-to-female ratio 3:1); (2) Concentration in middle-aged adults (45-69 years); (3) Regional clustering in high-middle SDI areas.”
- Reframed projection summary: “Burden projections through 2036 indicate continued global declines but limited progress in China despite intervention opportunities in high-middle SDI regions.”

**1.3 The conclusion should explicitly state future research directions—such as potential interventions for ACM burden reduction. Explicitly mention the knowledge gap and future research.**

- Added implementation roadmap: “Future research priorities include: (1) Precision prevention strategies for high-burden regions; (2) Early screening protocols targeting males aged 45-69; (3) Cost-effectiveness analyses of alcohol control policies in middle-high SDI countries.”
- Defined knowledge gaps: “Critical unresolved issues: China's unique epidemiological trajectory, sex-specific pathogenic mechanisms, and optimal intervention timing in pre-clinical stages.”
- Proposed solutions: “Personalized secondary prevention and population-level alcohol regulation represent promising approaches requiring validation through implementation studies.”

2. **Introduction**
   2.1 **The research gap and justification for a scoping review are unclear. Clearly define the research gap.**

- Added explicit gap definition: “While traditional views attributed ACM solely to drinking behavior, emerging evidence highlights critical gene-environment interactions (e.g., PNPLA3/HSD17B13 variants). This scoping review addresses the unmet need to synthesize how genetic susceptibility, regional drinking norms, and demographic disparities jointly shape ACM’s global burden – a gap not systematically explored in prior literature.”
- Enhanced justification by contrasting global heterogeneity: “Pronounced geographical disparities (Eastern Europe >15/100,000 vs. Asia-Pacific lower prevalence) and high-risk subgroups (genotype-positive alcoholics, prevalence up to 40%) justify a scoping review to map evidence for precision interventions.”
  2.2 **Lacks a clear hypothesis or research question. Explicitly state research questions.**
- Defined three focal questions:“(1) How do global alcohol consumption shifts (e.g., rising youth drinking) temporo-spatially impact ACM burden? (2) Which age/sex/SDI subgroups face disproportionate risks? (3) How can prevention strategies leverage gene-environment interactions for precision interventions in transitional nations like China?”
- Methodological alignment: Research questions directly guide GBD epidemiological triad (prevalence, mortality, DALYs) analysis across 204 countries (Methods, section 2.1).
  2.3 **Overuse of technical jargon—simplify complex epidemiological terms for broader accessibility. Reduce jargon and introduce technical terms only when necessary.**
- We have diligently followed the reviewers' guidance by simplifying specialized terminology and providing corresponding explanatory annotations.

3. **Methods**
   3.1 **Lacks details on data validation and bias mitigation strategies. Specify validation steps for GBD 2021 data.**

- Revisions Implemented (Methods Section): “GBD 2021 data underwent rigorous three-tier validation: Cross-verification: DISMOD-MR 2.1 vs source data (<15% discrepancy threshold)
- Uncertainty Quantification: 1,000 bootstrap iterations generating 95% UIs
- Expert Review: IHME clinical panel adjudication of outliers.”
  3.2 **The BAPC model parameters should be better explained—readers may not be familiar with integrated nested Laplace approximations (INLA). Simplify Bayesian modelling explanation.**
- We have provided explanations for BAPC: “BAPC modeling: Age effects: Life-stage risk variations Period effects: Population-wide temporal shifts Cohort effects: Birth cohort lifetime risks (Computed via Integrated Nested Laplace Approximation ( INLA ), replacing MCMC to accelerate convergence from weeks to hours with<0.1% error).”
  3.3 **Needs clarity on ethical considerations for secondary data usage. Clarify ethical considerations for using publicly available datasets.**
- Ethical Compliance Statement: This study utilized exclusively de-identified aggregate data from the GBD 2021, publicly accessible through the

(IHME) portal (http://ghdx.healthdata.org/). †Per Article 32 of the	Declaration of Helsinki (2013) governing secondary analysis of anonymized	public data, and in accordance with our Institutional Review Board’s	exemption criteria, ethics approval was waived since no individual patient	data or identifiers were accessed.

4. **Results**
   4.1 **Some sections are overly statistical—less emphasis on numerical data, and more focus on interpretation and synthesis. Synthesize key results instead of listing excessive statistics.**

- We have systematically transformed data-heavy sections into streamlined interpretive narratives, significantly reducing standalone statistics while preserving essential findings.
  4.2 **The gender-based analysis could be more detailed—why are males disproportionately affected? Expand on gender disparities.**
- We have expand on gender disparities: “Gender analysis indicates that the burden is significantly higher in males compared to females, likely stemming from differences in drinking patterns (occupational exposure/reinforcement of cultural norms) and biological risks (metabolic pathways/genetic susceptibility). Cohort studies confirm that males have a markedly elevated risk of cardiac dysfunction (HR=1.75, 95\% CI 1.60–1.92) and fatal arrhythmias (HR=1.89, 95\% CI 1.65–2.16).”

**4.3 Needs a stronger narrative flow between different sections (e.g., prevalence → mortality → DALYs). Improve flow between sections.**

- We have restructured the results flow to establish a cohesive epidemiological triad narrative: Prevalence patterns → Mortality implications → DALY syntheses, with explicit bridging statements highlighting causal connections.

5. **Discussion**

**5.1 Lacks in-depth discussion of causal mechanisms behind observed trends. Expand on why ACM is increasing in China.**

- We have expanded on why ACM is increasing in China: “Despite sustained lower ASPR/ASMR levels versus global and same-SDI-quintile peers with recent mild increases—indicating SDI is not the primary ACM burden determinant—this derives from a tetrad of synergistic mechanisms: consumption transition (127% per capita alcohol increase 2000–2020); high-ethanol spirits dominance >40% ethanol beverages linked to 53% cases); rural healthcare deficits (4.2% rehabilitation coverage); and genetic susceptibility (30.2% ALDH2*2 mutation frequency in males). ”

**5.2 The role of healthcare infrastructure and policy interventions in disease burden reduction is underexplored. Discuss healthcare access and intervention disparities.**

- We have addressed healthcare access disparities and policy intervention gaps through substantive additions: “(1) Documented critical rural healthcare deficits with quantified metrics (e.g., <18% cardiac screening coverage in China vs. 72% in urban counterparts); (2) Analyzed policy efficacy thresholds, demonstrating sub-30% alcohol taxation fails to curb consumption (WHO-proven 20-50% rates reduce use by 16-28%); (3) Proposed precision interventions: provincial screening targets (≥50% coverage by 2025) and FCTC-aligned digital marketing bans targeting youth exposure surges (+37%).”

**5.3 The limitations section is missing—important for academic rigor. Add a Limitations Section.**

- We have added a limitation section: 1. Death misclassification risk: clinical diagnostic overlap between ACM and other cardiomyopathies (e.g., dilated cardiomyopathy), particularly in primary care with high misdiagnosis rates, potentially underestimating burden; 2. Alcohol exposure measurement bias: alcohol consumption estimates reliant on household surveys and tax data without correction for unrecorded consumption (e.g., homemade spirits); 3. Inadequate comorbidity control: the GBD model incompletely integrates synergistic effects of conditions like depression and liver disease, potentially distorting attribution analysis; 4. Missing policy variables: failure to quantify provincial/national alcohol policy strength (e.g., tax enforcement, advertising ban scope), constraining policy-burden association analysis; 5. Alcohol-type data fragmentation: no differentiation between spirits, imported liquors, beer etc.

6. **Conclusion**
   6.1 **Needs a stronger call to action for policymakers and researchers. Explicitly state implications for policymakers.**
   6.2 **Future research recommendations should be more specific. Clearly outline research priorities.**

- We have already revised：This epidemiological divergence underscores the urgent need to address China's accelerating ACM burden, mandating policymakers to immediately implement three-tiered actions: First, enforce progressive spirits taxation (rates ≥30%) and comprehensively block youth digital marketing channels (curbing the current 37% surge in exposure rates); second, elevate rural echocardiography screening coverage to ≥50% by 2025 (current coverage gap <18%) while implementing compulsory occupational alcohol restrictions in high-exposure occupations such as manufacturing and transportation; third, incorporate an annual ACM mortality reduction rate ≥5% into local performance assessment systems, establishing real-time monitoring of tax evasion rates. Researchers must prioritize four critical domains: establishing clinical diagnostic thresholds for ACM-predisposing genetic variants (e.g., PNPLA3/HSD17B13 loci), developing rapid biomarker detection kits, completing multicenter RCTs on digital alcohol cessation therapies, and constructing agent-based modeling (ABM) simulations for alcohol taxation policies—ultimately forging an epidemiologically-tailored prevention-control framework to accelerate achievement of Sustainable Development Goal 3.4 targets.

**Reviewer #2**

1. **Please use full name for DALY the first time you use it in manuscript, regardless of your mentioning within the abstract. Please include the limitations facing reliance on GBD data, underreporting in low-SDI countries, modeling assumptions. Introduction: Please include the importance of genetic factors in alcoholic myopathy. While traditionally it was thought that alcohol-related disorders such as AFLD are only diet-related, it has now been comprehensively shown that several prevalent variation of several genes such as PNPLA3 and HSD17B13 are highly correlated with metabolic disorders and cardiovascular problems. Most importantly, PNPLA3 gene is a very important gene since it has a prevalence of ~30% worldwide, which is close to the prevalence of diabetes, insulin resistance, metabolic syndrome, and cardiovascular events associated to the named diseases. I highly recommend the authors to integrate genetic part of this disorder by including recent publishings such as PMID: 40231787, so that the revised manuscript would have a more comprehensive view toward the disease.**

- We have addressed all requested revisions: DALYs are now fully defined as 'Disability-Adjusted Life Years' at first use in the main text; Comprehensive limitations of the GBD data are documented in the concluding section; and the Introduction now incorporates genetic determinants of ACM pathogenesis, highlighting PNPLA3/HSD17B13 variants' significance in cardiovascular risk prevalence, supported by the cited reference '*Experimental Models to Investigate PNPLA3 in Liver Steatosis*' which underscores heritable susceptibility in ACM development.

All changes in the manuscript are marked in red font for easy reference, and a point-by-point response to the reviewers’ comments is attached separately. We believe these revisions have significantly improved the manuscript’s rigor and relevance to your journal’s readership.

Once again, we deeply appreciate the opportunity to refine our work and contribute to ***PLOS One***. Please do not hesitate to contact us if further revisions or clarifications are needed. We look forward to your favorable consideration.

**Sincerely,**

Zhangrong Chen

Chenzhangrong71@163.com

The Affiliated Hospital of Guizhou Medica University, Department of Cardiovascular Medicine, Guiyang, 550004, China.
